# *N*-Benzoyl-*N*′,*N*′-dimethyl­thio­urea

**DOI:** 10.1107/S1600536811005137

**Published:** 2011-02-16

**Authors:** Hiram Pérez, Rodrigo S. Corrêa, Ana María Plutín, Anislay Álvarez, Yvonne Mascarenhas

**Affiliations:** a Departamento de Química Inorgánica, Facultad de Química, Universidad de la Habana, Habana 10400, Cuba; b Departamento de Química, Universidade Federal de São Carlos, CEP 13565-905, São Carlos, SP, Brazil; c Laboratorio de Síntesis Orgánica, Facultad de Química, Universidad de la Habana, Habana 10400, Cuba; d Grupo de Cristalografia, Instituto de Fisica de São Carlos, Universidade de São Paulo, CEP 13560-970, São Carlos, Brazil

## Abstract

In the title compound, C_10_H_12_N_2_OS, the amide NCO group is twisted relative to the thio­ureido SCN_2_ group, forming a dihedral angle of 55.3 (2)°. The crystal packing shows inter­molecular N—H⋯S and weak C—H⋯O inter­actions, the former giving rise to the formation of centrosymmetric *R*_2_^2^(8) dimers.

## Related literature

For general background to *N*-acyl-*N′*,*N′*-disubstituted thio­urea, see: Koch (2001 ▶); Sosa-Albertus & Piris (2001 ▶); Pérez *et al.* (2008*a* ▶). For related structures, see: Arslan *et al.* (2003 ▶); Bolte & Fink (2003 ▶); Pérez *et al.* (2008*b* ▶); Gomes *et al.* (2010 ▶). For details of the synthesis, see: Nagasawa & Mitsunobu (1981 ▶); Che *et al.* (1999 ▶). For graph-set notation, see: Bernstein *et al.* (1995 ▶).
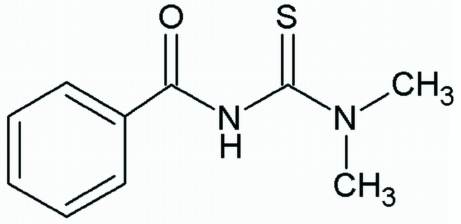


## Experimental

### 

#### Crystal data

C_10_H_12_N_2_OS
*M* *_r_* = 208.28
Monoclinic, 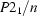

*a* = 10.8602 (9) Å
*b* = 5.5590 (6) Å
*c* = 18.6864 (10) Å
β = 102.768 (5)°
*V* = 1100.24 (16) Å^3^
*Z* = 4
Mo *K*α radiation
μ = 0.26 mm^−1^
*T* = 294 K
0.26 × 0.13 × 0.13 mm

#### Data collection

Nonius KappaCCD diffractometer
Absorption correction: gaussian (Coppens et al., 1965 ▶) *T*_min_ = 0.943, *T*_max_ = 0.969
7078 measured reflections
2282 independent reflections
1762 reflections with *I* > 2σ(*I*)
*R*_int_ = 0.041

#### Refinement

*R*[*F* ^2^ > 2σ(*F* ^2^)] = 0.043
*wR*(*F* ^2^) = 0.120
*S* = 1.05
2282 reflections
133 parameters
H atoms treated by a mixture of independent and constrained refinement
Δρ_max_ = 0.17 e Å^−3^
Δρ_min_ = −0.27 e Å^−3^

### 

Data collection: *COLLECT* (Enraf–Nonius, 2000 ▶); cell refinement: *SCALEPACK* (Otwinowski & Minor 1997 ▶); data reduction: *DENZO* (Otwinowski & Minor 1997 ▶) and *SCALEPACK*; program(s) used to solve structure: *SHELXS97* (Sheldrick, 2008 ▶); program(s) used to refine structure: *SHELXL97* (Sheldrick, 2008 ▶); molecular graphics: *ORTEP-3 for Windows* (Farrugia, 1997 ▶) and *Mercury* (Macrae *et al.*, 2006 ▶); software used to prepare material for publication: *WinGX* (Farrugia, 1999 ▶).

## Supplementary Material

Crystal structure: contains datablocks global, I. DOI: 10.1107/S1600536811005137/gk2336sup1.cif

Structure factors: contains datablocks I. DOI: 10.1107/S1600536811005137/gk2336Isup2.hkl

Additional supplementary materials:  crystallographic information; 3D view; checkCIF report

## Figures and Tables

**Table 1:** Hydrogen-bond geometry (Å, °)

*D*—H⋯*A*	*D*—H	H⋯*A*	*D*⋯*A*	*D*—H⋯*A*
N1—H1⋯S1^i^	0.85 (2)	2.65 (2)	3.4335 (17)	154.2 (18)
C10—H10*C*⋯O1^ii^	0.96	2.38	3.265 (3)	153

Symmetry codes: (i) 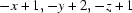; (ii) 
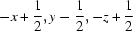
.

**Fig. 1.**
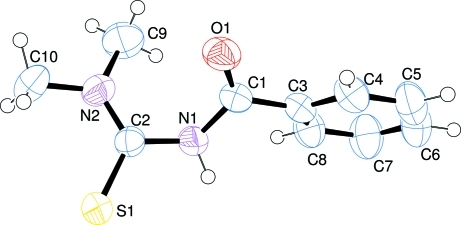
The molecular structure of the title compound. Displacement ellipsoids are drawn at the 50% probability level.

**Fig. 2.**
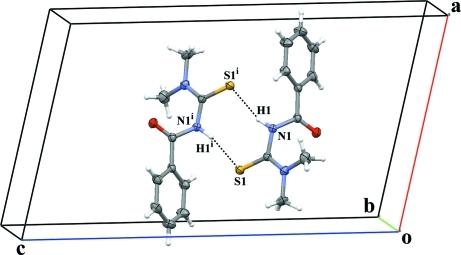
The R22(8) centrosymmetric dimer lying across the centre of symmetry at (1/2,0, 1/2). Hydrogen bonds are shown as dashed lines [symmetry code (i) -x + 1, -y + 2, -z + 1].

## supplementary crystallographic information

### Comment

*N*-Acyl-*N'*,*N'*-disubstituted thiourea derivatives have been a subject of investigations due to their ability to form stable metal complexes (Koch *et al.*, 2001). The crystal structure analysis of the title compound was undertaken as a continuation of our interest in these *N'*,*N'*-disubstituted acylthiourea derivatives as intermediates towards novel heterocycles and for the systematic study of their bioactivity and complexation behavior (Pérez *et al.*, 2008*a*). On the other hand, the crystal structure determination of this compound helps to confirm its most stable molecular conformation, previously predicted by theoretical methods (Sosa-Albertus & Piris, 2001) in order to explain the behavior of polydentate systems in alkylation reactions.

The main bond lengths of the title compound are within the ranges obtained for similar compounds (Pérez *et al.*, 2008*b*; Arslan *et al.*, 2003). The C–S and C–O bonds both show the expected double-bond character. However, the C–N bonds of acylthioureido fragment are intermediate between those expected for single and double C–N bonds (1.47 and 1.27 Å, respectively). These results can be explained by the existence of resonance in this part of the molecule. The conformation with respect to the thiocarbonyl and carbonyl groups is twisted, as reflected by the torsion angles O1/C1/N1/C2 and C1/N1/C2/N2 of -2.6 (3) and 57.9 (2)°. The dihedral angle between the O1/C1/N1 and S1/C2/N2 planes is 55.3 (2)°, while that between the O1/C1/N1 plane and the benzene ring is 35.8 (2)°. Compared to the diethyl analog (Bolte & Fink, 2003) and its monoclinic polymorph (Gomes, *et al.*, 2010), the molecular confomation of the title molecule is significantly less twisted, as reflected by the corresponding torsion angles O/C/N/C [12.48 (4)°, in Bolte & Fink (2003); 7.58 (17)° in Gomes *et al.* (2010) and C/N/C/N (-80.79 (3)° in Bolte & Fink (2003); -71.44 (14)° in Gomes *et al. (*2010)]. The dihedral angle between the O/C/N and S/C/N planes is also smaller than those of the diethyl analog [(73.9 (2)° in Bolte & Fink (2003); 67.3 (1)° in Gomes *et al.* (2010)]. In the crystal structure (Fig. 2), an N—H···S(-*x* + 1,-*y* + 2,-*z* + 1) hydrogen bond links the molecules into *R*^2^_2_(8) centrosymetric dimers (Bernstein *et al.*, 1995) across the crystallographic centre of symmetry at (1/2, 0, 1/2). The molecules are also linked by weak C—H···O hydrogen bonds (Table 1).

### Experimental

*N*-Benzoyl-*N'*,*N'*-dimethylthiourea was prepared using the standard procedure previously reported in the literature (Nagasawa & Mitsunobu, 1981) by the reaction of benzoyl chloride with KSCN in anhydrous acetone, and then condensation with dimethylamine. The synthesis of title compound was previously reported (Che *et al.*, 1999). Recrystallization from acetone/water solution (1:1, *v*/*v*) yielded colourless crystals (1.6 g, 7.5 mmol, 75%). m.p. 448 K. Analysis calculated for C_10_H_12_N_2_OS: C 57.67, H 5.80, N 13.45, S 15.40%. Found: C 57.88, H 5.92, N 13.60, S 15.19%.

### Refinement

H atoms bonded to C atoms were included in calculated positions and refined as riding, with C–H = 0.93 or 0.96 Å and with *U*_iso_(H) = 1.2Ueq(C) or 1.5Ueq(C). H atom bonded to N atom was located in difference Fourier synthesis and was refined isotropically.

### Crystal data

**Table:**

C_10_H_12_N_2_OS	*F*(000) = 440
*M**_r_* = 208.28	*D*_x_ = 1.257 Mg m^−^^3^
Monoclinic, *P*2_1_/*n*	Mo *K*α radiation, λ = 0.71073 Å
Hall symbol: -P 2yn	Cell parameters from 1912 reflections
*a* = 10.8602 (9) Å	θ = 3.5–26.7°
*b* = 5.5590 (6) Å	µ = 0.26 mm^−^^1^
*c* = 18.6864 (10) Å	*T* = 294 K
β = 102.768 (5)°	Prism, colourless
*V* = 1100.24 (16) Å^3^	0.26 × 0.13 × 0.13 mm
*Z* = 4	

### Data collection

**Table:**

Nonius KappaCCD diffractometer	1762 reflections with *I* > 2σ(*I*)
ω scan	*R*_int_ = 0.041
Absorption correction: gaussian (Coppens et al., 1965)	θ_max_ = 26.7°, θ_min_ = 3.5°
*T*_min_ = 0.943, *T*_max_ = 0.969	*h* = −13→13
7078 measured reflections	*k* = −6→7
2282 independent reflections	*l* = −22→23

### Refinement

**Table:**

Refinement on *F*^2^	0 restraints
Least-squares matrix: full	H atoms treated by a mixture of independent and constrained refinement
*R*[*F*^2^ > 2σ(*F*^2^)] = 0.043	*w* = 1/[σ^2^(*F*_o_^2^) + (0.0587*P*)^2^ + 0.1899*P*] where *P* = (*F*_o_^2^ + 2*F*_c_^2^)/3
*wR*(*F*^2^) = 0.120	(Δ/σ)_max_ < 0.001
*S* = 1.05	Δρ_max_ = 0.17 e Å^−^^3^
2282 reflections	Δρ_min_ = −0.27 e Å^−^^3^
133 parameters	

### Special details

**Table:**

Geometry. All e.s.d.'s (except the e.s.d. in the dihedral angle between two l.s. planes) are estimated using the full covariance matrix. The cell e.s.d.'s are taken into account individually in the estimation of e.s.d.'s in distances, angles and torsion angles; correlations between e.s.d.'s in cell parameters are only used when they are defined by crystal symmetry. An approximate (isotropic) treatment of cell e.s.d.'s is used for estimating e.s.d.'s involving l.s. planes.

### Fractional atomic coordinates and isotropic or equivalent isotropic displacement parameters (Å^2^)

**Table:**

	*x*	*y*	*z*	*U*_iso_*/*U*_eq_	
C1	0.55382 (16)	0.7917 (3)	0.35288 (9)	0.0475 (4)	
C2	0.38501 (16)	0.6859 (3)	0.41573 (8)	0.0459 (4)	
C3	0.69073 (16)	0.8469 (3)	0.36576 (9)	0.0484 (4)	
C4	0.7314 (2)	1.0217 (4)	0.32357 (10)	0.0638 (5)	
H4	0.6731	1.103	0.2878	0.077*	
C5	0.8583 (2)	1.0756 (5)	0.33449 (13)	0.0778 (6)	
H5	0.8853	1.1951	0.3067	0.093*	
C6	0.9442 (2)	0.9537 (5)	0.38608 (14)	0.0822 (7)	
H6	1.0296	0.9899	0.393	0.099*	
C7	0.90569 (19)	0.7787 (5)	0.42771 (14)	0.0785 (6)	
H7	0.965	0.6965	0.4627	0.094*	
C8	0.77898 (18)	0.7235 (4)	0.41797 (11)	0.0608 (5)	
H8	0.7529	0.6042	0.4463	0.073*	
C9	0.3971 (3)	0.3391 (4)	0.33595 (14)	0.0806 (7)	
H9A	0.4844	0.3341	0.3609	0.121*	
H9B	0.3906	0.3891	0.2861	0.121*	
H9C	0.3607	0.182	0.3366	0.121*	
C10	0.19432 (19)	0.4648 (5)	0.36413 (12)	0.0753 (6)	
H10A	0.1503	0.6153	0.3619	0.113*	
H10B	0.1803	0.373	0.4051	0.113*	
H10C	0.1636	0.3765	0.3196	0.113*	
N1	0.51125 (13)	0.7326 (3)	0.41555 (8)	0.0485 (4)	
N2	0.32963 (14)	0.5100 (3)	0.37299 (8)	0.0557 (4)	
O1	0.48381 (12)	0.7991 (3)	0.29255 (6)	0.0623 (4)	
S1	0.31430 (4)	0.84543 (10)	0.47135 (3)	0.0602 (2)	
H1	0.5514 (19)	0.802 (4)	0.4545 (11)	0.066 (6)*	

### Atomic displacement parameters (Å^2^)

**Table:**

	*U*^11^	*U*^22^	*U*^33^	*U*^12^	*U*^13^	*U*^23^
C1	0.0508 (9)	0.0491 (10)	0.0440 (8)	0.0049 (7)	0.0133 (7)	−0.0020 (7)
C2	0.0449 (9)	0.0501 (10)	0.0416 (8)	−0.0028 (7)	0.0073 (6)	0.0012 (7)
C3	0.0505 (9)	0.0528 (10)	0.0452 (8)	0.0041 (7)	0.0176 (7)	−0.0013 (7)
C4	0.0672 (12)	0.0686 (13)	0.0596 (11)	0.0016 (10)	0.0228 (9)	0.0091 (9)
C5	0.0744 (14)	0.0826 (15)	0.0854 (14)	−0.0127 (12)	0.0369 (12)	0.0106 (13)
C6	0.0530 (12)	0.0933 (18)	0.1055 (17)	−0.0082 (11)	0.0288 (12)	0.0052 (15)
C7	0.0476 (11)	0.0905 (16)	0.0961 (16)	0.0084 (11)	0.0130 (11)	0.0168 (13)
C8	0.0541 (11)	0.0630 (12)	0.0669 (11)	0.0047 (9)	0.0170 (9)	0.0115 (9)
C9	0.0980 (17)	0.0543 (12)	0.0940 (16)	−0.0025 (11)	0.0312 (14)	−0.0231 (11)
C10	0.0614 (12)	0.0833 (16)	0.0749 (13)	−0.0227 (11)	0.0017 (10)	−0.0145 (11)
N1	0.0435 (8)	0.0610 (9)	0.0410 (7)	−0.0033 (6)	0.0093 (6)	−0.0038 (7)
N2	0.0561 (9)	0.0519 (9)	0.0582 (8)	−0.0062 (7)	0.0106 (7)	−0.0106 (7)
O1	0.0590 (8)	0.0824 (10)	0.0436 (7)	0.0056 (6)	0.0075 (6)	0.0021 (6)
S1	0.0504 (3)	0.0722 (4)	0.0626 (3)	−0.0118 (2)	0.0222 (2)	−0.0184 (2)

### Geometric parameters (Å, °)

**Table:**

C1—O1	1.2133 (19)	C6—H6	0.93
C1—N1	1.390 (2)	C7—C8	1.382 (3)
C1—C3	1.485 (2)	C7—H7	0.93
C2—N2	1.321 (2)	C8—H8	0.93
C2—N1	1.396 (2)	C9—N2	1.464 (3)
C2—S1	1.6759 (17)	C9—H9A	0.96
C3—C4	1.384 (3)	C9—H9B	0.96
C3—C8	1.388 (3)	C9—H9C	0.96
C4—C5	1.381 (3)	C10—N2	1.464 (2)
C4—H4	0.93	C10—H10A	0.96
C5—C6	1.364 (3)	C10—H10B	0.96
C5—H5	0.93	C10—H10C	0.96
C6—C7	1.368 (3)	N1—H1	0.85 (2)
			
O1—C1—N1	122.28 (16)	C7—C8—C3	119.70 (19)
O1—C1—C3	122.89 (15)	C7—C8—H8	120.1
N1—C1—C3	114.83 (14)	C3—C8—H8	120.1
N2—C2—N1	116.90 (15)	N2—C9—H9A	109.5
N2—C2—S1	123.85 (14)	N2—C9—H9B	109.5
N1—C2—S1	119.21 (12)	H9A—C9—H9B	109.5
C4—C3—C8	119.31 (17)	N2—C9—H9C	109.5
C4—C3—C1	119.15 (16)	H9A—C9—H9C	109.5
C8—C3—C1	121.52 (16)	H9B—C9—H9C	109.5
C5—C4—C3	120.14 (19)	N2—C10—H10A	109.5
C5—C4—H4	119.9	N2—C10—H10B	109.5
C3—C4—H4	119.9	H10A—C10—H10B	109.5
C6—C5—C4	120.0 (2)	N2—C10—H10C	109.5
C6—C5—H5	120	H10A—C10—H10C	109.5
C4—C5—H5	120	H10B—C10—H10C	109.5
C5—C6—C7	120.5 (2)	C1—N1—C2	123.76 (14)
C5—C6—H6	119.7	C1—N1—H1	114.1 (14)
C7—C6—H6	119.7	C2—N1—H1	113.5 (14)
C6—C7—C8	120.3 (2)	C2—N2—C10	120.48 (16)
C6—C7—H7	119.9	C2—N2—C9	123.91 (17)
C8—C7—H7	119.9	C10—N2—C9	115.48 (17)
			
O1—C1—C3—C4	34.5 (3)	C4—C3—C8—C7	0.8 (3)
N1—C1—C3—C4	−144.94 (17)	C1—C3—C8—C7	179.32 (19)
O1—C1—C3—C8	−144.04 (19)	O1—C1—N1—C2	−2.6 (3)
N1—C1—C3—C8	36.5 (2)	C3—C1—N1—C2	176.89 (15)
C8—C3—C4—C5	−1.3 (3)	N2—C2—N1—C1	57.9 (2)
C1—C3—C4—C5	−179.87 (19)	S1—C2—N1—C1	−124.37 (16)
C3—C4—C5—C6	1.1 (3)	N1—C2—N2—C10	−173.79 (17)
C4—C5—C6—C7	−0.4 (4)	S1—C2—N2—C10	8.6 (2)
C5—C6—C7—C8	−0.1 (4)	N1—C2—N2—C9	10.6 (3)
C6—C7—C8—C3	−0.1 (4)	S1—C2—N2—C9	−167.03 (16)

### Hydrogen-bond geometry (Å, °)

**Table:**

*D*—H···*A*	*D*—H	H···*A*	*D*···*A*	*D*—H···*A*
N1—H1···S1^i^	0.85 (2)	2.65 (2)	3.4335 (17)	154.2 (18)
C9—H9A···N1	0.96	2.43	2.778 (3)	101
C9—H9B···O1	0.96	2.49	2.902 (3)	106
C10—H10C···O1^ii^	0.96	2.38	3.265 (3)	153

Symmetry codes: (i) −*x*+1, −*y*+2, −*z*+1; (ii) −*x*+1/2, *y*−1/2, −*z*+1/2.
